# Chronic vs. Acute Interactions between Supraoptic Oxytocin Neurons and Astrocytes during Lactation: Role of Glial Fibrillary Acidic Protein Plasticity

**DOI:** 10.1100/tsw.2009.148

**Published:** 2009-11-18

**Authors:** Yu-Feng Wang, Kathryn A. Hamilton

**Affiliations:** Department of Cellular Biology and Anatomy, Louisiana State University Health Sciences Center, Shreveport, USA

**Keywords:** burst firing, cytoskeleton, glia, hypothalamo-neurohypophysial system, milk ejection reflex, neuroendocrine system, rhythmic activity, suckling

## Abstract

In this article, we review studies of astrocytic-neuronal interactions and their effects on the activity of oxytocin (OXT) neurons within the magnocellular hypothalamo-neurohypophysial system. Previous work over several decades has shown that withdrawal of astrocyte processes increases OXT neuron excitability in the hypothalamic supraoptic nucleus (SON) during lactation. However, chronically disabling astrocyte withdrawal does not significantly affect the functioning of OXT neurons during suckling. Nevertheless, acute changes in a cytoskeletal element of astrocytes, glial fibrillary acidic protein (GFAP), occur in concert with changes in OXT neuronal activity during suckling. Here, we compare these changes in GFAP and related proteins with chronic changes that persist throughout lactation. During lactation, a decrease in GFAP levels accompanies retraction of astrocyte processes surrounding OXT neurons in the SON, resulting from high extracellular levels of OXT. During the initial stage of suckling, acute increases in OXT levels further strengthen this GFAP reduction and facilitate the retraction of astrocyte processes. This change, in turn, facilitates burst discharges of OXT neurons and leads to a transient increase in excitatory neurochemicals. This transient neurochemical surge acts to reverse GFAP expression and results in postburst inhibition of OXT neurons. The acute changes in astrocyte GFAP levels seen during suckling likely recur periodically, accompanied by rhythmic changes in glutamate metabolism, water transport, gliotransmitter release, and spatial relationships between astrocytes and OXT neurons. In the neurohypophysis, astrocyte retraction and reversal with accompanying GFAP plasticity also likely occur during lactation and suckling, which facilitates OXT release coordinated with its action in the SON. These studies of the dynamic interactions that occur between astrocytes and OXT neurons mediated by GFAP extend our understanding of astrocyte functions within the central nervous system.

Morphological and functional plasticity of astrocytes plays an important role in modulating neuronal activity. In a variety of brain regions, astrocyte plasticity has been shown to influence synaptic transmission[1], learning and memory consolidation[2], epilepsy[3], and reproductive neuroendocrine functions[4]. This plasticity is influenced by both neural and humoral factors. Astrocytes can sense and react to changes in the external environment via purinergic receptors[1], $K^+$ channels[5], glutamate transporters[6], and water channels[7]. In turn, they contribute to the homeostasis of the local environment via uptake of ions and neurotransmitters, degrading or converting neurochemicals, volume transport, and providing metabolic substances and neurotransmitter or its precursors to neurons. Changes in neuronal activity that affect the local neurochemical environment inevitably affect astrocytes, which in turn modulate neuronal activity and output. Thus, astrocyte plasticity and neuronal activity are interconnected processes. Here, we review interactions between astrocytes and oxytocin (OXT) neurons during the entire period of lactation (chronic phase) and during a bout of suckling (acute phase). We focus on acute changes in glial fibrillary acidic protein (GFAP, an astrocyte cytoskeletal element) and its interaction with associated molecules that affect the functioning of the magnocellular hypothalamo-neurohypophysial system during suckling.

## CHRONIC INTERACTIONS BETWEEN ASTROCYTES AND OXT NEURONS

The influence of astrocytes on neuronal activity largely depends on changes in astrocyte morphology and functions that are induced by neuronal activity. These changes have been particularly well documented during parturition and lactation in the supraoptic nucleus (SON) of the hypothalamus in rodents. The chronic morphological changes were recently reviewed[8] and will only be summarized here.

In the SON, astrocyte processes surround magnocellular neurons. During the late stage of pregnancy and lactation, astrocyte processes, together with their GFAP content, withdraw from the OXT neurons[9], which increases direct neuronal apposition. This change facilitates formation of gap junctions and therefore rapid communication between apposed neurons. It also facilitates formation of additional chemical synapses on OXT neurons from glutamatergic and GABAergic neurons[10,11]. The astrocyte processes that surround neurons actively transport $K^+$ and glutamate[12], and maintain low extracellular $K^+$ and glutamate levels. In addition, astrocytes secrete enzymes that metabolize neuromodulators. For example, astrocyte ecto-5'nucleotidase in the SON[13] converts ATP to adenosine. It is therefore likely that astrocyte retraction in the SON results in increased levels of extracellular $K^+$, glutamate, and other neurochemicals, and in increased neuronal coupling[14,15], which together facilitate the coordinated burst firing of magnocellular neurons[16]. This burst firing, in turn, maximizes release of OXT from the neurohypophysis[17], which triggers milk ejection by eliciting contraction of the mammary myoepithelium. These observations have guided studies of the hypothalamo-neurohypophysial system over several decades, which have concluded that astrocyte retraction during late pregnancy and lactation is essential in order to synchronize activation of OXT neurons and ensure milk ejection[8,18].

Astrocyte retraction during lactation is closely related to chronic changes that occur in the neurochemical environment around term. Before term, the responsiveness of OXT neurons is restrained by progesterone[19], which potentiates GABA-mediated inhibition[20]. As parturition approaches, this inhibition fades as progesterone secretion collapses; instead, a central opioid inhibitory mechanism is activated that restrains excitation of OXT neurons. During parturition, progesterone and β-endophin levels decrease[21], intranuclear OXT secretion increases[22], and OXT receptor (OXTR) expression also increases[23], eliciting GFAP reduction/retraction of astrocyte processes. In addition, norepinephrine levels increase well before the onset of delivery, dopamine release increases during the second half of parturition, and a bolus of glutamate is released immediately before delivery[24]. Norepinephrine and dopamine activate β-adrenergic receptors[25] and $D_2$ dopamine receptors[26], respectively, which act to reduce GFAP levels. At the same time, the effect of OXT on GFAP expression is enhanced by the increased estrogen and glutamate levels[27,28] that occur during parturition. In the SON, the increased OXT levels appear to maintain low GFAP expression throughout lactation, until estrogen release is restored near weaning. Either alone or with increased progesterone levels, the increased estrogen levels act to increase GFAP expression[29].

It was previously shown that polysialic acid moieties of neural cell adhesion molecules are required for astrocyte retraction in the SON during lactation[30]. Recently, however, the importance of these moieties and of astrocyte morphological plasticity to the milk ejection reflex has been questioned. Catheline et al.[31] showed that hypothalamic microinjections of endoneuraminidase did not eliminate activation of OXT neurons or milk ejection during suckling. Endoneuraminidase removes the polysialic acid moieties and blocks astrocyte withdrawal in the SON of pregnant rats. The failure of endoneuraminidase to block activation of OXT neurons therefore suggests that chronic retraction of astrocyte processes is not essential to astrocyte-mediated modulation of OXT neuronal activity.

## ACUTE INTERACTIONS BETWEEN ASTROCYTES AND OXT NEURONS

The most important events during lactation are suckling of offspring and milk-ejection reflex. If astrocyte plasticity is critical for neuroendocrine modulation of OXT neuronal activity during lactation, then suckling stimulation should elicit changes in the astrocytes of the SON. In examining potential changes in astrocyte plasticity, it is important to keep in mind that OXT neuronal activity in the SON is highly dynamic during suckling. Wakerley and Lincoln[32] originally showed that the firing rate of OXT neurons increases 20- to 40-fold immediately before OXT release from the neurohypophysis. The discharges consist of 30- to 80-Hz spike bursts lasting 1–4 sec each, which recur every 4–8 min[33]. The bursts are also spatiotemporally synchronized among OXT neurons[16]. A brief inhibitory period follows each burst (postburst inhibition), then firing activity of the OXT neurons increases gradually until the next burst[34]. This dynamic OXT neuronal activity pattern cannot be explained by a simple model emphasizing a single phase of astrocyte process retraction during suckling. Nor can a simple chronic model explain the finding that astrocyte plasticity is critical to OXT neuronal activity, which is highly dynamic[35].

A limited number of studies have focused on acute, reversible changes in astrocyte morphology that can be triggered by OXT treatment or hypertonic stimulation. Water deprivation for 4–24 h causes retraction of the astrocyte process in rat SON, which is similar to the retraction seen following chronic (10-day) dehydration and during lactation[36]. Hypertonic stimulation of cultured rat hypothalamic astrocytes induces synthesis and release of glutamate and taurine within as little as 10 min[37]. These studies show that altering the osmotic or neurochemical environment can acutely affect astrocyte morphology. Moreover, in SON slices, OXT (0.1 $\mu M$, 1–4 h) can induce withdrawal of astrocyte processes[27] if the environment is also hyperosmotic. It is known that suckling causes a long-lasting increase in OXT levels in the SON, and that OXT is a critical factor regulating the bursts of OXT neurons as well as the milk-ejection reflex[38]. Thus, these findings also suggest that suckling stimulates acute, dynamic changes in astrocyte morphology and function that occur in conjunction with the acute, dynamic changes in OXT neuronal activity described above.

Recent studies provide strong evidence for the importance of astrocytes in facilitating burst firing of OXT neurons. During suckling, astrocyte processes retract from OXT neurons before the first milk ejection[39] and they remain retracted until shortly after the bursts. The retraction permits increases in the OXT, glutamate, and $K^+$ levels in the extracellular space. It also permits formation of gap junctions among OXT neurons, which facilitates synchronized bursting. The bursting activity, in turn, changes the local neurochemical environment, resulting in a rapid increase in extracellular $K^+$[40] and, likely, bolus release of OXT, together with corelease of glutamate[41] and ATP[42]. After each burst, astrocyte processes actively uptake $K^+$, together with water from the extracellular space. The astrocytes convert ATP to adenosine[13], and peptidase from neurons hydrolyzes OXT[43]. Together, these changes in the extracellular milieu appear to result in re-expansion of astrocyte processes that serves to suppress OXT neuronal activity. ATP and high $K^+$[44] can also stimulate astrocytes to release taurine, which can also suppress OXT neuronal activity. The recurrence of this cycle of retraction and expansion guarantees rhythmic, synchronous bursting of OXT neurons, and therefore ensures intermittent milk ejection.

A question that immediately comes to mind is whether or not OXT levels change in the SON as a result of bursting. A gradual increase in OXT levels can induce either excitation or inhibition of OXT neurons depending on the time course and dose of OXT applied. Bath application of OXT to SON slices can simulate the gradual increase in OXT levels that occurs during suckling[22]. This increases the OXT neuronal firing rate within the first 5–15 min, after which the excitatory effect of OXT on firing activity gradually declines, and may even turn into a period of silence[45,46]. Although OXT levels in the SON are elevated for at least 30 min[22], each cycle of bursts and milk ejection lasts only for a few minutes. This discrepancy suggests the possibility that the OXT level or OXTR sensitivity transiently decreases following each burst. Alternatively, other factors must act to overcome the influence of persistent high OXT levels throughout suckling. As presented in the following sections, we postulate that acute, dynamic changes in the expression of GFAP and related molecules play a major role in regulating astrocyte morphological and functional plasticity, and therefore OXT neuronal activity during suckling.

## GFAP PLASTICITY DURING SUCKLING AND THE UNDERLYING MECHANISMS

GFAP is an intermediate filament that is composed of a head, rod, and a tail domain[47]. The overall size of GFAP filaments depends on the extent of assembly of different domains and polymerization of single strands (monomers). Depolymerization of GFAP filaments typically produces GFAP monomers, dimmers, and tetramers. In addition, GFAP monomers can be degraded to GFAP domain fragments. In astrocytes, GFAP is usually bound to other cytoskeletal components to form the visible GFAP filaments that shape astrocyte morphology. Thus, changes in GFAP distribution mirror astrocyte morphology. Classically, astrocyte morphological plasticity has been studied by examining GFAP immunoreactivity using transmission electron microscopy. However, GFAP is often absent from fine astrocyte processes that surround synapses and neuronal somata[8]. In the studies reviewed here, we therefore examined GFAP distribution and levels in the SON using alternative methods (confocal microscopy and Western blotting) following suckling and after pharmacological simulation of SON slices[35].

We first observed effects of suckling on GFAP expression in the SON of lactating rats[35]. In these studies, we separated lactating rats from 10 pups for 4 h, and then divided the dams into three groups according to the known regulation of the milk-ejection reflex[48]. The groups were as follows: dams that had not suckled their pups (nonsuckling group); dams that had suckled their pups for 5–10 min, but had not experienced the first milk-ejection reflex (nonmilk-ejection group); dams that had suckled their pups until the third or fourth milk ejection had occurred (milk-ejection group). SON samples from dams that were allowed to suckle before the first milk ejection would have experienced the electrical and secretory activity of OXT neurons and related changes in astrocytes before and in between OXT neuron bursts and milk ejections. Within 1 min following a burst, OXT neurons can again be ready to generate a spike burst[49]. Dams that were allowed to suckle until the third or fourth milk-ejection reflex would therefore have experienced stable, rhythmic OXT neuron bursting[34,50]. With this classification scheme, it is therefore possible to examine the full dynamic range of the milk-ejection reflex and how astrocyte plasticity depends on OXT neuron bursting in relation to the reflex. This classification of suckling stages differs from previous classifications, which only included suckling and nonsuckling groups[22,24,51]. Our results showed that suckling causes a significant reduction in GFAP immunostaining and levels concomitant with retraction of astrocyte processes and enlargement of astrocyte somata. These changes appear to result from the translocation or redistribution of GFAP filaments from the processes to the somata of the astrocytes.

Further analysis showed that the reduced GFAP immunolabeling in the SON was strongest within the dorsal portion, where OXT neurons are the dominant neuronal subtype. Similar regional changes in GFAP labeling have previously been observed during lactation[52] and in response to dehydration[53]. The change in GFAP labeling in the SON is consistent with the realignment of radial glia-like processes from primarily vertical to primarily lateral orientation[54]. As discussed above, GFAP dynamic organization determines astrocyte morphology[47] and influences OXT neuronal activity[8,18]. It therefore seems likely that the GFAP reduction seen during the early stage of suckling should play the same role in the milk-ejection reflex as it does in response to chronic stimuli that increase extracellular $K^+$, glutamate and OXT levels, synaptic innervation, and junctional communication between OXT neurons[55]. The major difference between the acute changes in GFAP and astrocyte morphology and previously reported chronic changes lies in compensatory mechanisms[56] that occur in response to chronic stimuli, but is not in response to acute disabling of astrocyte plasticity using L-aminoadipic acid or fluorocitrate[35].

Suckling stimulation causes milk ejections intermittently. It is therefore likely that changes in GFAP also occur intermittently. Our results showed, in fact, that decreased immunolabeling for GFAP filaments and for the 50-kDa GFAP monomer in the SON during the initial stage of suckling reversely increased immediately after the third or fourth milk ejection[35]. The immunolabeling was significantly greater in terms of the number of GFAP filaments and overall intensity of GFAP labeling. Astrocyte somata were also larger in slices from milk-ejection than from nonsuckling dams; however, the intensity of GFAP staining in the somata following the milk ejection was reduced in comparison with the intensity seen in samples from nonmilk-ejection suckling dams. These findings leave little question about the dynamic nature of GFAP plasticity in response to acute stimulation by brief suckling.

Parturition[57] and suckling[32] both activate OXT neuron bursts, which are temporally closely related to release of OXT[22], noradrenaline[51], and glutamate[24] in the hypothalamus. Increased noradrenaline[10] and OXT[39] levels *in vitro* cause astrocyte retraction. Glutamatergic innervation is also essential for OXT effects on GFAP plasticity[27]. Increased glutamate levels maintain OXT neuronal excitability[46] and burst firing[58]. In SON slices, both OXT[58] and $\alpha_1$-adrenergic receptor agonist[59,60] also induce burst firing of OXT neurons. However, the increased OXT level is the most dramatic neurochemical change during suckling[22], and it triggers burst discharges of OXT neurons in both brain slices[58] and dams during suckling[61].

In addition to the neurochemical changes that occur during suckling, a transient increase in extracellular $K^+$ also occurs in the hypothalamo-neurohypophysial system following each OXT neuron burst[40]. We therefore tested effects of OXT and transient elevation of extracellular $K^+$ on GFAP plasticity in SON slices[35]. We found that bath delivery of 10 p*M* to 1 n*M* OXT for 30 min reduced GFAP levels and triggered a GFAP redistribution that was identical to the redistribution evoked by suckling. Reduced GFAP immunolabeling of astrocyte processes appeared to result from depolymerization and decomposition of GFAP filaments. Blocking proteolysis using protease inhibitors did not block the OXT-evoked decrease in GFAP immunolabeling, but did block the reduced expression of the 50-kDa GFAP protein. Moreover, the reversed GFAP expression following bursts and milk ejection was mimicked by exposing SON slices to 12 m*M* $K^+$ for 30 sec in the presence of OXT. These results provide strong evidence that increased $K^+$ levels (and likely increased ATP and glutamate levels as well) similar to those that occur during suckling could play an important role in the reversal of GFAP immunolabeling and levels that follow bursts.

## ROLE OF GFAP PLASTICITY IN DYNAMIC ASTROCYTE-OXT NEURONAL INTERACTIONS

An essential question concerning GFAP plasticity during suckling is whether the reversal of GFAP immunolabeling and levels is accompanied with re-expansion of astrocyte processes. No obvious expansion of astrocyte processes was observed following the milk ejection in our previous study. This finding was based on immunostaining of filamentous actin and then observing apposition of SON neurons[39]. The discrepancy between the GFAP labeling/levels and immunolabeling for actin can be explained in two ways. First, a transient expansion of astrocyte processes could have occurred in our GFAP study, which was not detected. Considering that the burst discharges of OXT neurons are transient events and that the postburst inhibitory phase has a short duration[33], it is quite possible that astrocyte processes expanded and then quickly retracted before samples were taken. The second explanation is that the astrocyte processes expanded following the bursts, but the expansion was too small to be resolved by confocal microscopy. This latter possibility is supported by the finding that astrocyte somata remain large following bursts and milk ejection; this observation makes it seem unlikely that a large volume of cytosol was transported from the soma to the processes within tens of seconds. Moreover, if no rapid bulk transport of cytosol occurred, then what is the functional implication of the changes in GFAP immunolabeling and levels that we observed during suckling, and why does disabling withdrawal of astrocyte processes fail to disrupt the milk-ejection reflex[31]?

To answer these questions, we confirmed that astrocyte plasticity is essential for the milk-ejection reflex. A glial toxin, L-aminoadipic acid or fluorocitrate, was delivered to the SON of lactating rat dams using an intracerebroventricular catheter[35]. We then confirmed that synchronized burst discharge in OXT neurons was eliminated as indicated by the low milk availability to the litter and small changes in the intramammary pressure during suckling. We found that gliotoxin injections reduced rather than increased GFAP expression. The observation that GFAP expression reduced without facilitating the milk-ejection reflex suggests that the influence of GFAP on OXT neuronal functions cannot merely be attributed to changes in astrocyte morphology.

In astrocytes, L-aminoadipic acid inhibits glutamine synthetase and $Na^+$-dependent glutamate reuptake[62], and fluorocitrate acts indirectly via multiple functional proteins to inhibit glial energy metabolism[63]. We therefore examined the association of GFAP with a series of astrocyte proteins[35] that have been implicated in astrocyte functions. In our previous immunostaining studies, we had determined that certain proteins colocalized with GFAP-positive regions of astrocytes. They included OXTR and cyclooxygenase 2[64], G protein α and β subunits[58], phosphorylated extracellular signal-regulated protein kinase 1/2 (pERK1/2), and filamentous actin[39]. Morphological colocalization suggests, but cannot confirm, direct molecular interactions. We therefore performed a series of coimmunoprecipitation experiments. After immunoprecipitating GFAP, we detected other proteins using Western blots. The results showed that actin, aquaporin-4 (a water channel protein), and glutamine synthetase are all dynamically associated with GFAP during suckling. At the initial stage of suckling, molecular association between GFAP and aquaporin-4 temporarily decreases and then the association increases following milk ejection. These changes suggest that in the SON, GFAP controls astrocyte morphological plasticity by changing water transportation through the plasma membrane, as occurs in astrocytes of other brain areas[65]. Suckling also caused an increase and then decrease in the association of GFAP with actin. Increased interaction of GFAP with actin during the initial stage of suckling could function to pull the plasma membrane of astrocyte processes toward the soma, away from the OXT neurons. By contrast, following burst discharges, decreased interaction of GFAP with actin could function to release the plasma membrane from the spatial barrier effect of the cytoskeleton, thereby facilitating astrocyte release of gliotransmitters. Finally, suckling decreased the association between GFAP and glutamine synthetase. An essential role of astrocytes in synaptic transmission is to provide glutamine to neurons following glutamate uptake; astrocyte glutamine synthetase serves this purpose by converting glutamate into glutamine[66]. Following the OXT neuron bursts, dissociation of glutamine synthetase from GFAP in astrocytes should accelerate glutamate metabolism by allowing the enzyme to access the submembrane region where uptake occurs and to convert glutamate to glutamine rapidly. We propose that GFAP functions both in changing astrocyte morphology and in localizing proteins that are important to astrocyte functions. This proposal is consistent with results of others showing that GFAP is molecularly associated with glutamate transporters[67] and is responsible for trafficking the transporters to the plasma membrane[68]. Thus, regardless of whether or not astrocyte processes retract from OXT neurons during lactation[31] or re-expansion following the burst during suckling[39], GFAP plasticity could still affect the burst discharges of OXT neurons by influencing glutamate metabolism and the local neurochemical milieu.

In addition to these roles, GFAP might also play a role in burst generation by affecting the release of taurine[69], glutamate[41], and ATP[70] from astrocytes in the SON. OXT stimulates taurine efflux from cultured pituicytes[71] and taurine inhibits SON neuronal activity[72], likely via extrasynaptic glycine receptors[73]. Because hypotonicity-evoked taurine release is causally related to GFAP expression[74], the changes in GFAP expression that occur during suckling might first reduce and then increase taurine release. Taurine release requires higher OXT than vasopressin (VP) levels[71]. Thus, taurine might only be effective following burst discharges. Firing activity and glutamate trigger release of ATP[75] and glutamate[76] from astrocytes, which further facilitates burst discharges. In turn, increased ATP and glutamate following bursts can act to increase GFAP levels[77,78]. A transient increase in GFAP might facilitate insertion of excitatory amino acid transporter 2, glutamate-aspartate transporter, and taurine transporter on astrocyte plasma membranes. These transporters could then act to reduce extracellular levels of amino acid transmitters. This very likely occurs in the SON, because knocking out GFAP increases extracellular glutamate levels[79]. GFAP has been shown to facilitate membrane insertion of excitatory amino acid transporter in astrocytes[68]. Astrocytes can quickly reduce high ATP levels by converting ATP to adenosine via ecto-5'nucleotidase, which has been shown to be expressed in the SON[13]. Adenosine is known to inhibit OXT neuronal activity[80]. It is possible that this enzyme is also transported to the plasma membrane following GFAP extension.

Together, these observations lead us to propose the following model of astrocyte plasticity during lactation (Fig. 1). From pregnancy to lactation, OXT-evoked GFAP depolymerization and chronic retraction of astrocyte processes permits establishment of a relatively excitatory neurochemical environment for OXT neuron bursting in response to suckling stimulation (Fig. 1A and B). The vertically oriented astrocyte processes are interposed between neighboring OXT neurons; they occur close to the neuronal somata and most of the dendrites. As a result, they are more readily influenced by OXT released from OXT neurons in comparison with astrocyte somata and proximal processes. During suckling and before the bursts (Fig. 1B and C), glutamate and ATP released from astrocytes, together with OXT-evoked GFAP depolymerization and process retraction, facilitate the bursting of OXT neurons. However, at this time, gliotransmitter levels are not high enough to reverse OXT-reduced GFAP levels and distribution. Following the bursts (Fig. 1C and D), a bulk release of glutamate and ATP from astrocytes triggered by bolus release of neurochemicals from OXT neurons causes polymerization of GFAP filaments. Excessive glutamate activates metabolic glutamate receptors, while increasing endocytosis of excitatory receptors of OXT neurons. The GFAP increase facilitates astrocyte uptake of glutamate, $K^+$, and water, which leads to release of taurine and activation of glycine receptors on OXT neurons. Consequently, OXT firing activity is suppressed, accounting for the postburst inhibition. At the same time, excessive glutamate is converted to glutamine, increasing the glutamine pool for synthesis of glutamate at presynaptic terminals and increasing excitation of OXT neurons. Following bursts, it is also possible that excitatory receptors are reinserted on OXT neuron plasma membranes, increasing neuronal sensitivity to excitatory neurochemicals. Excessive taurine would also be taken up by astrocytes. As a result, OXT neuronal excitability would increase again as a new milk-ejection reflex begins. Lastly, it should be mentioned that GABA plays an important role in the milk-ejection reflex. During lactation, GABAergic innervation increases significantly[10,81]. Importantly, increased GABA levels increase GFAP levels[26]. Because GABA levels are relatively stable in the SON throughout suckling[82], it likely that GABA exerts a tonic inhibitory effect on OXT neuronal activity by increasing GFAP levels.

## CHRONIC AND ACUTE PLASTICITY IN NEUROHYPOPHYSIAL ASTROCYTES

Lactation and suckling modulate both OXT and VP secretion within the neurohypophysis. Neurohypophysial astrocytes (pituicytes) interact with magnocellular neuron terminals dynamically during lactation and dehydration[10]. Before pregnancy/lactation, pituicytes engulf neuronal terminals and interpose their processes between OXT neuronal terminals and the basement membranes of blood vessels, reducing the release of OXT into the blood driven by firing activity of magnocellular neurons[83,84]. During lactation and chronic dehydration, pituicyte processes retract from these areas[85], allowing the neuronal terminals direct access to the perivascular space and facilitating OXT and VP release

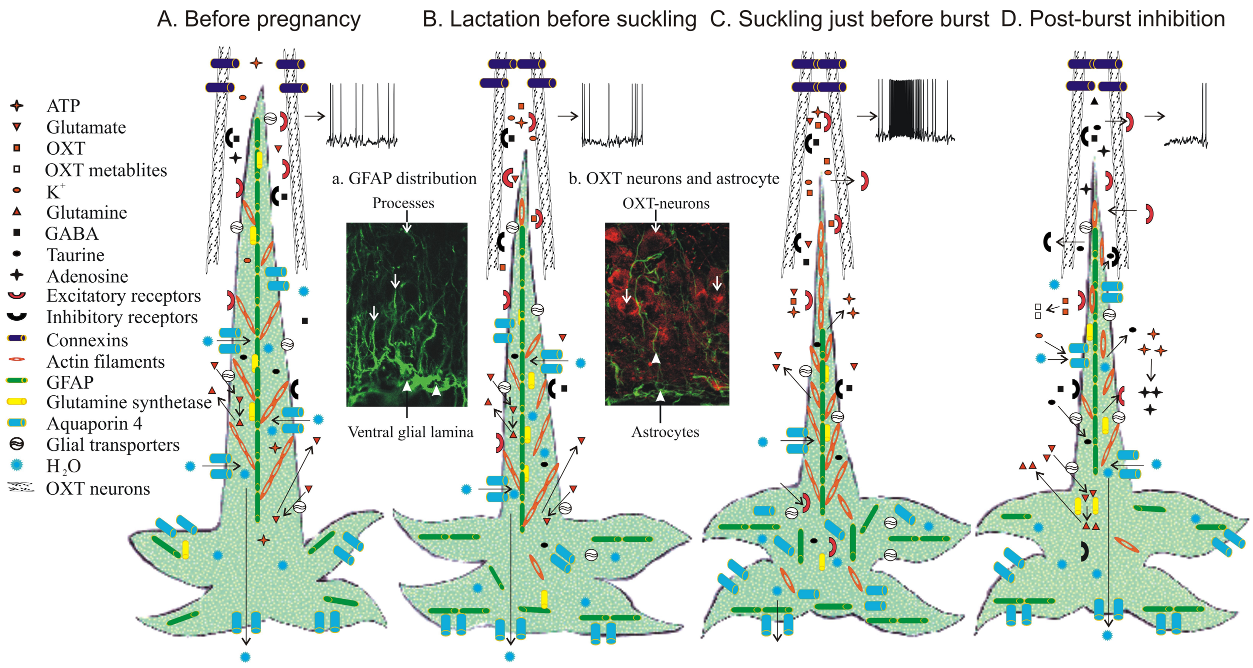

**FIGURE 1.** Mechanisms underlying chronic and acute stages of GFAP plasticity and astrocyte-OXT neuron interactions in the SON. An astrocyte cell body and a vertical process extending dorsally from ventral glial lamina are shown in green. The vertical process is interposed between plasma membranes of two OXT neurons (dashed lines). (A–D) GFAP plasticity before pregnancy (A); during lactation, but before suckling (B); during suckling, but before the first milk ejection (C); and immediately following the third or fourth milk ejection during suckling (D). Inset "a" shows confocal image of GFAP distribution (in green) in the SON: white arrowheads, ventral glial lamina-located astrocyte somata; white arrows, vertically oriented astrocyte processes. Inset "b" shows dorsally located OXT-neurophysin–positive OXT neurons (white arrows, red) and they are interposed by GFAP-positive astrocyte processes (arrowheads, green). (A) GFAP levels and distribution are dynamically related to neurotransmitter/modulator levels, receptor expression, levels of amino acid transporters, water channel expression, and associated $K^+$ permeability, membrane localization and activity of intracellular enzymes, and actin cytoskeleton dynamics. Before and during the early stage of pregnancy, GFAP metabolism and the factors are shown in a state of dynamic balance, which permits continuous firing of OXT neurons. (B) Following the reduction in sex steroid hormones and increases in OXT levels that occur in the SON around parturition, GFAP levels are reduced in vertically orientated astrocyte processes, and increased in the somata. In this state, increased extracellular levels of OXT and OXTR trigger breakdown of GFAP and retraction of the interposed astrocyte processes, leading to apposition of OXT neuronal membranes, gap junctional communication, and increased chemical synaptic innervation. (C) Brief suckling stimulation increases OXT secretion and results in further depolymerization and decomposition of GFAP in the interposed processes, volume expansion of somata and proximal processes, and secondary release of ATP and glutamate from astrocytes. Together with intracellular mechanisms proposed previously (see review by Hatton and Wang[48]), these factors trigger burst discharges of OXT neurons. During this process, receptors of excitatory agents are gradually desensitized. (D) Each burst discharge triggers bolus release of ATP and glutamate from OXT neurons as well as astrocytes; in astrocytes, GFAP reduction reverses and amino acid transporters are inserted, leading to decreases in extracellular glutamate and $K^+$, reduction of junctional communication, increases in adenosine and taurine levels, and activation of metabolic glutamate and glycine receptors. This produces a period of silence following the bursts. At the same time, more glutamine will be made available by glutamine synthetase activity, taurine will be taken up, and OXT neuronal sensitivity to OXT and glutamate will be restored. Together, this results in gradual excitation of OXT neurons and retraction of the apposed astrocyte process accompanied by redistribution of GFAP.

into the blood via fenestrated capillaries. Acute as well as chronic changes in pituicyte morphology are observed during lactation in intact animals[86], in neurohypophysial explants[25,87] in response to osmotic stimulation and noradrenaline[25], and can even be induced in cultured pituicytes by activating β-adrenoceptors[88]. The major difference between chronic and acute pituicyte plasticity is that proliferation and cell death occur in response to chronic stimulation[89,90], but have not been observed in response to acute stimulation.

Mechanisms underlying the morphological plasticity of pituicytes have been reviewed recently[91]. Adrenaline, endotheline, taurine, VP and OXT, ATP and adenosine, nitric oxide, and other neurochemicals are involved in pituicyte retraction. The involvement of taurine is particularly relevant to this review. OXT increases taurine release from pituicytes via V1a receptor activation[71], and terminals of OXT neurons express taurine-susceptible glycine receptor[37]. It seems plausible that changes in pituicyte GFAP should resemble that seen in SON astrocytes, which as described above could modulate OXT release via actions of taurine. However, effects of taurine on OXT release likely depend on the functional state of the neurohypophysis and on the intensity of stimulation. A high taurine level would be required to increase OXT and VP release under basal conditions[92], whereas a low level would be required to suppress high $K^+$-evoked VP secretion[37].

As with plasticity of other types of astrocytes, pituicyte plasticity depends on cytoskeletal elements. GFAP has been identified in protoplasmic, but not fibrous, pituicytes, and GFAP reduction is typically observed accompanying pituicyte retraction in response to osmotic challenge and during lactation[93,94]. Acute changes in suckling-associated neurochemicals also regulate GFAP plasticity in pituicytes. In general, effects of the neurochemicals on GFAP plasticity are consistent with their effects on OXT secretion. Pituicytes are directly innervated by GABAergic neurons, and GABA acts to increase pituicyte GFAP[26] and to reduce OXT secretion[95]. The neurohypophysis also receives noradrenergic innervation[96]. Activation of the β-adrenergic receptor causes GFAP depolymerization and pituicyte retraction[88], and it increases OXT release[97]. Actions of the dopaminergic innervation of the neurohypophysis are less well understood. Activation of $D_2$ receptors reduces magnocellular neuronal activity[98] and OXT secretion[97]. This seems contrary to the effects of dopamine in reducing pituitary GFAP expression[26]. This inconsistency suggests that potential facilitatory effects of pituicyte retraction on OXT release are contingent on OXT neuronal activity. When OXT neuronal activity is inhibited, the potential for OXT release is enhanced in the neurohypophysis, yet no release occurs.

It remains to be determined whether suckling directly induces GFAP plasticity in pituicytes. Preliminary observations nevertheless suggest this possibility. In response to adenosine, rat pituicytes in primary culture assume stellate shapes instead of their normal flattened shapes; however, subsequent OXT stimulation causes them to flatten[99]. Before suckling stimulation, OXT release through the neurohypophysis does not change significantly. However, following burst discharges of OXT neurons, bolus OXT release occurs while extracellular $K^+$ increases in the neurohypophysis[40], both of which likely influences pituicyte morphology. Re-expansion of pituicyte processes[99] will quickly wrap OXT neuronal terminals and increase the distance between the terminals and blood vessels. Together with OXT-increased taurine release[71], these structural changes should decrease or terminate OXT release. Thus, it is very likely that similar changes in GFAP occur synergistically in the SON and in the neurohypophysis during suckling, although the time course and underlying mechanisms might differ to some extent.

## CONCLUSIONS

Different models have different advantages and disadvantages for studying chronic vs. acute changes in astrocytes and effects of the changes on neuronal activity. Dehydration and lactation are the two main models for studying chronic astrocyte morphological plasticity in the SON; both induce chronic retraction of astrocyte processes and associated changes in GFAP distribution and levels. The disadvantage of these chronic models is that they do not take into account dynamic astrocytic-neuronal interactions. Astrocyte cell lines and cultures afford better visibility and accessibility for studying cellular responses to environmental changes in response to both chronic and acute stimuli. However, *in vitro* preparations lack three-dimensional structural features of intact tissues that are important to normal physiological functions. The SON slice model offers other disadvantages, as well as advantages, for studying astrocyte-neuronal plasticity in the neural system. The disadvantages include poor image quality due to the slice thickness and the difficulty of identifying specific cellular sources of proteins due to the complexity of the tissue. Using an integrated approach (patch-clamp recording, confocal and electron microscopy, Western blotting, and coimmunoprecipitation), it is nevertheless possible to detect dynamic interactions between astrocytes and OXT neurons in slices that simulate the acute physiological changes observed in intact animals. Thus, it is possible to examine astrocytic-neuronal interactions *in vitro*, time-locked to different stages of the milk-ejection reflex. Although much recent progress has been made in understanding glialneuronal interactions in the SON, many important questions remain. We still do not know how neuronal signals trigger astrocyte GFAP plasticity, and why the somata and processes respond differently to the same stimulation. Nor do we know how plasticity of GFAP is linked to changes in activity of other proteins that influence astrocyte metabolism and functions. Similarly, the release patterns of neurochemicals from astrocytes as well as from structurally associated neurons have not been fully examined, but they seem critical to GFAP plasticity. Finally, we do not know if it might be possible to modulate astrocyte plasticity selectively by reversibly altering GFAP metabolism and organization *in vivo*. Our studies of the dynamic interactions that occur between astrocytes and OXT neurons via GFAP during suckling have provided a new arena in which to investigate these questions.

## ACKNOWLEDGMENT

This review is dedicated to Dr. Glenn I. Hatton. Portions of the work presented here were supported by NIH grants NS009140 to G.I. Hatton and DC007876 to K.A. Hamilton. We thank reviewers for advice on the manuscript.
